# Association between the atherogenic index of plasma and acute kidney injury in sepsis patients

**DOI:** 10.1371/journal.pone.0344477

**Published:** 2026-03-10

**Authors:** Rong Shuai, Lizhong Lin, Xianhua Zeng, Li Zhang

**Affiliations:** 1 Department of Laboratory Medicine, Changde Hospital, Xiangya School of Medicine, Central South University (The first people’s hospital of Changde city), Changde, Hunan, China; 2 Emergency ICU Unit, Changde Hospital, Xiangya School of Medicine, Central South University (The first people’s hospital of Changde city), Changde, Hunan, China; Stanford University School of Medicine, UNITED STATES OF AMERICA

## Abstract

**Background:**

Although metabolic abnormalities are directly linked to acute kidney injury (AKI) in septic patients, the function of the atherogenic index of plasma (AIP) is yet unknown.

**Objectives:**

This study examined the relationship between AIP and AKI risk in septic patients, providing insight into the role of lipid metabolism in renal injury.

**Methods:**

This study investigated patients with sepsis using data from the eICU Collaborative Research Database (eICU-CRD). The AIP was calculated as the base-10 logarithm of the ratio of triglycerides to high-density lipoprotein cholesterol. We employed multivariate logistic regression to evaluate the association between AIP and AKI. Potential nonlinear relationships were assessed using restricted cubic spline (RCS) curve modeling. Additionally, subgroup and sensitivity analyses were conducted to assess the robustness of these findings.

**Results:**

The median age of the 771 patients was 66.6 years, with 173 (22.4%) developing AKI. A positive linear association was observed between the AIP and the risk of AKI. Specifically, each 0.1-unit increase in AIP was associated with a 10% higher risk of AKI (OR = 1.10, 95% CI: 1.05–1.15, P < 0.001). Sensitivity analyses after excluding patients with renal infections showed that AIP remained associated with the risk of acute kidney injury. Stratified analyses showed no significant interactions (all P for interaction > 0.05) in gender, age, BMI, SOFA score, GCS score, presence of other diseases (diabetes mellitus, chronic obstructive pulmonary disease, congestive heart failure).

**Conclusions:**

For septic patients, the relationship between AIP and risk of AKI was positively linear, with higher AIP indicating higher risk of AKI.

## Introduction

Sepsis remains a leading cause of mortality worldwide [1–3] and is a major precipitant of acute kidney injury (AKI) in critically ill patients [4,5], driven by systemic inflammation [6,7], endothelial dysfunction [8–10], and metabolic disturbances [11–13]. Although recent advances have deepened our understanding of sepsis-associated organ injury [14–17], the contribution of lipid metabolism dysregulation to AKI pathogenesis remains poorly understood. The Atherogenic Index of Plasma (AIP)—defined as the logarithm of the triglyceride to high-density lipoprotein cholesterol ratio (log[TG/HDL-C])—has emerged as a biomarker reflecting lipid-driven inflammation and endothelial impairment, with established associations to cardiovascular and renal diseases [18,19]. Nevertheless, its relationship with AKI risk in the setting of sepsis, where metabolic imbalances and oxidative stress are exacerbated, has yet to be systematically explored.

The pathophysiological mechanisms linking AIP to sepsis-associated AKI stem from its dual role as a marker of atherogenic dyslipidemia and systemic inflammation. Elevated AIP correlates with enhanced oxidative stress, endothelial injury, and proinflammatory cytokine release, processes that exacerbate renal microvascular damage and tubular cell apoptosis during sepsis [13,20]. Studies in non-septic populations demonstrate associations between AIP and acute kidney injury (AKI) severity [18] as well as chronic kidney disease (CKD) progression [21]. Sepsis-specific lipid toxicity and immunometabolic crosstalk may further amplify AIP’s detrimental effects on renal function [12,22–24], providing a theoretical basis for investigating AIP-AKI associations. However, no large-scale clinical studies have validated this hypothesis.

This study aims to systematically investigate the quantitative relationship between the AIP and AKI in a cohort of 771 sepsis patients from the eICU database. We hypothesize that AIP, as a biomarker of dysregulated lipid metabolism and systemic inflammation, is significantly associated with AKI risk in sepsis. Using multivariable-adjusted models, we will evaluate the independent effects of both continuous AIP increments and categorical exposure on AKI development. These findings are expected to advance our understanding of lipid metabolic disturbances in sepsis-associated renal injury and provide a theoretical foundation for potential therapeutic interventions.

## Methodology

### Study design and data source

A retrospective cohort study was conducted using data from the eICU Collaborative Research Database (eICU-CRD) [25], a multicenter intensive care unit repository containing comprehensive clinical records of over 200,000 ICU admissions across the United States between 2014 and 2015. The eICU-CRD, sourced from the Philips Healthcare eICU program, includes a broad spectrum of patient-level clinical variables. To protect patient confidentiality, this study adhered to the Health Insurance Portability and Accountability Act (HIPAA) Safe Harbor provisions, qualifying it for exemption from ethical review by the Massachusetts Institute of Technology Institutional Review Board [26]. The study was conducted in accordance with the ethical principles of the Declaration of Helsinki and complied with all relevant guidelines and regulations. Author Li Zhang completed the requisite training and was granted access to the eICU database (record ID: 68930432).

### Study population

**Fig 1:**
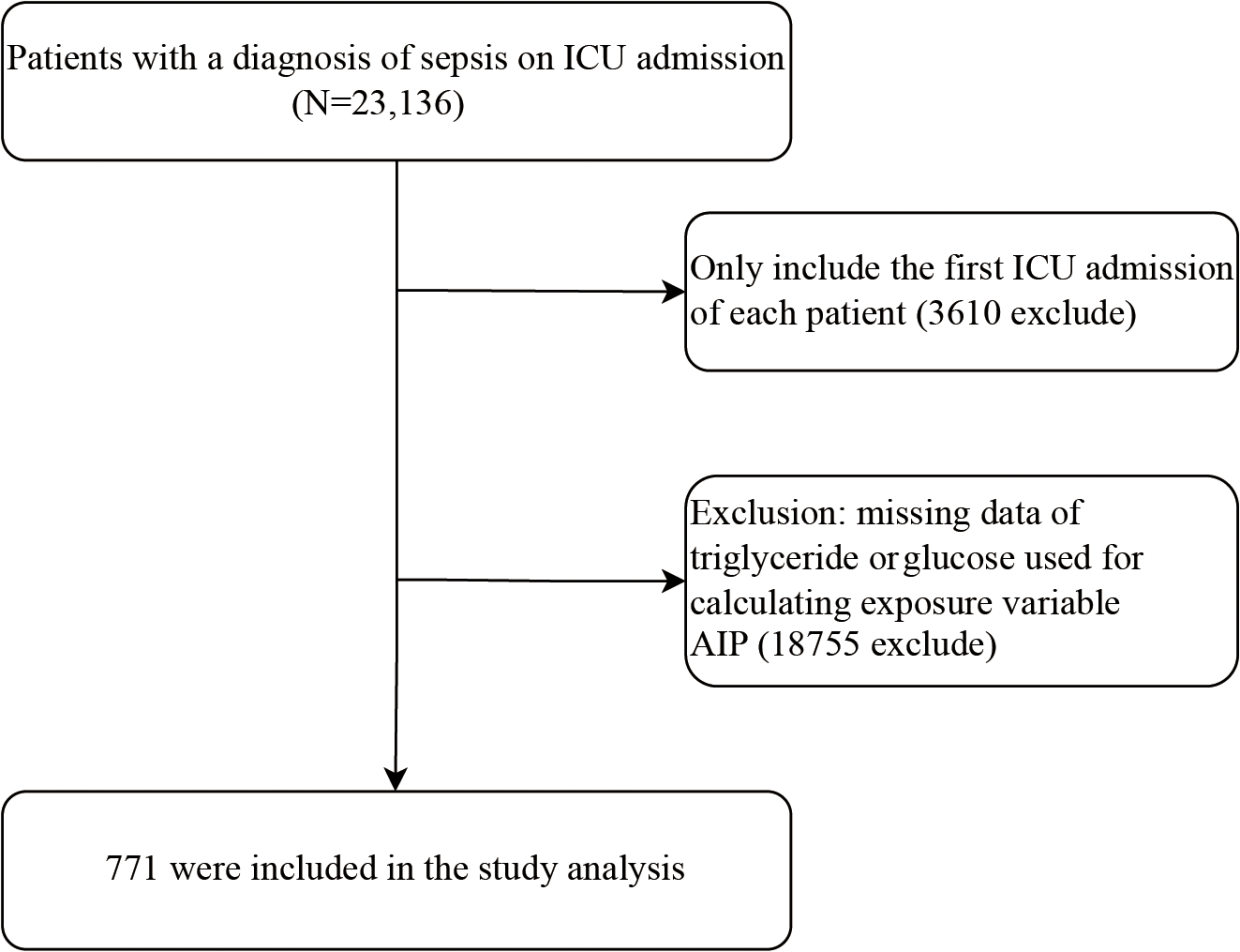
Flowchart of study participants.

Patients diagnosed with sepsis at ICU admission were included in this study. Sepsis was defined according to the Sepsis-3 criteria, requiring a documented or suspected infection accompanied by an acute increase of 2 or more points in the Sequential Organ Failure Assessment (SOFA) score [27]. Infection status was confirmed using ICD-9 codes extracted from the eICU Collaborative Research Database. Exclusion criteria were: non-first ICU admissions and missing post-admission triglyceride or high-density lipoprotein cholesterol (HDL-C) measurements. The participant selection workflow is detailed in Fig 1.

### Data collection and missing data

The eICU database contains demographic data, physiological measurements from point-of-care monitors, diagnoses coded using the International Classification of Diseases, 9th Revision, Clinical Modification (ICD-9-CM), and various laboratory results collected during routine clinical care. For this study, we extracted physiological variables, baseline characteristics, laboratory indicators, and comorbidity information. Physiological parameters—including body temperature, heart rate, and mean arterial pressure—were obtained from the Apache ApsVar table, while SOFA and Glasgow Coma Scale (GCS) scores were calculated accordingly. Baseline characteristics comprised age, sex, race, body mass index (BMI), white blood cell count, urea, creatinine, total cholesterol (TC), triglycerides, high-density lipoprotein cholesterol, and comorbidities such as chronic obstructive pulmonary disease (COPD), congestive heart failure (CHF), acute myocardial infarction (AMI), diabetes mellitus (DM), and AKI. The data completeness for these variables was high but not uniform. Data for the following variables were complete, with no missing values (0%): Gender, Age, Sepsis admission, COPD, CHF, AMI, DM, and SOFA score. Several other variables had missing values: Ethnicity (0.78% missing), BMI (3.24% missing), Heart rate and Mean BP (0.65% missing for each), Acute physiology score and APACHE score (10.38% missing for each), GCS score and TC (1.82% missing for each), and WBC (4.67% missing).

### Ethical approval

The data for this study were extracted from the eICU-CRD in accordance with the PhysioNet Review Board's Data Use Protocol (Record ID: 68930432). Given that this study was based on anonymised data and did not involve direct patient intervention, it was exempt from ethical approval.

### Exposure variables

The exposure variable was the Atherogenic Index of Plasma (AIP), calculated as log10(TG/HDL-C) [18]. The timing of AIP measurement (upon ICU admission) preceded the assessment of AKI (within 48 hours after admission), establishing a temporal sequence for the analysis. We analyzed AIP as a continuous variable to explore the association between every 0.1unit change in AIP (AIP*10) and acute kidney injury. Patients were divided into 3 groups based on AIP tertiles: T1 (−0.697 to 0.017, n = 257), T2 (0.017 to 0.363, n = 257), T3 (0.364 to 2.204, n = 257).

### Outcome variables

The primary outcome was acute kidney injury (AKI) after ICU admission. The lowest serum creatinine (SCr) value within 7 days before admission was used as the baseline SCr [28]. When SCr was missing before admission, the SCr first measured at admission was used as the baseline SCr. The stage of AKI was defined according to the maximum SCr value obtained within 48 hours after admission. Acute kidney injury was defined as an SCr greater than or equal to 3.0 times the baseline SCr or an increase in SCr concentration of no less than 4.0 mg/dL or initiation of renal replacement therapy [29]. The secondary outcome was the duration of hospital stay.

### Statistical analysis

The AIP was divided into three groups, with continuous variables expressed as mean ± SD or median and interquartile range (IQR). Categorical variables were expressed as frequencies and percentages. Comparison of variability between groups variables were analysed using one-way ANOVA Kruskal-Wallis test for skewed distribution for normally distributed variables and chi-square test for categorical variables. Restricted cubic spline (RCS) regression was used to analyse the dose-response relationship between AIP and AKI in patients with sepsis. We used logistic regression models to estimate the relationship between AIP and AKI after adjusting for confounders. The results were expressed as odds ratios (OR) and 95% confidence intervals (95% CI). We used a comprehensive approach to identify risk factors associated with ARF from clinical expertise, original studies, and existing literature. Considering the above factors, covariates included gender, age, ethnicity, BMI, Heart rate, Mean BP, WBC, TC, Sepsis admission, GCS score, SOFA score, COPD, CHF, AMI and DM. To assess the robustness of the findings, sensitivity analyses were performed. We analyzed whether the relationship between AIP and AKI was stable after the expulsion of participants whose initial infection was in the kidney. For further exploratory analyses, subgroup homogeneity was assessed using a stratified logistic regression model, taking into account sex, age, BMI, SOFA score, GCS score, and diabetes, COPD, and CHF.

## Results

### Characteristics of participants

Table 1 summarizes the baseline characteristics of 771 participants stratified by AIP tertiles (T1: −0.697 to 0.017; T2: 0.017 to 0.363; T3: 0.364 to 2.204). Significant differences (P < 0.05) were observed across groups in age, BMI, heart rate, renal function markers (BUN, creatinine), triglyceride levels, GCS score, SOFA score, and select comorbidities including diabetes mellitus and acute kidney injury. Notably, patients in the highest tertile (T3) were younger (62.8 ± 15.4 years), had a higher BMI (32.3 ± 10.6 kg/m²), elevated heart rates (116.4 ± 28.3 bpm), markedly increased triglyceride levels (258.1 ± 393.0 mg/dL), and the highest incidence of acute kidney injury (33.9%). The prevalence of diabetes was also significantly greater in T3 (22.2%) compared to T1 (14.0%) and T2 (12.8%). These data indicate that elevated AIP is associated with metabolic derangements and increased organ dysfunction among patients with sepsis.

**Table 1:** Baseline characteristics of participants.

Parameters	Total (n = 771)	AIP tertiles			*P* value
		T1(−0.697 ~ 0.017) (n = 257)	T2(0.017 ~ 0.363) (n = 257	T3(−0.364 ~ 2.204) (n = 257)	
**Demographics**					
Gender, n (%)					0.411
Male	369 (47.9)	124 (48.2)	115 (44.7)	130 (50.6)	
Female	402 (52.1)	133 (51.8)	142 (55.3)	127 (49.4)	
Age (years)	66.6 ± 15.1	69.2 ± 15.6	67.8 ± 13.6	62.8 ± 15.4	< 0.001
Ethnicity, n (%)					0.442
Caucasian	544 (70.6)	178 (69.3)	181 (70.4)	185 (72)	
African American	89 (11.5)	35 (13.6)	33 (12.8)	21 (8.2)	
Hispanic	73 (9.5)	26 (10.1)	25 (9.7)	22 (8.6)	
Asian	40 (5.2)	10 (3.9)	10 (3.9)	20 (7.8)	
Native American	5 (0.6)	1 (0.4)	3 (1.2)	1 (0.4)	
Other/Unknown	20 (2.6)	7 (2.7)	5 (2.0)	8 (3.0)	
BMI(kg/m²)	30.4 ± 9.8	28.2 ± 8.5	30.6 ± 9.7	32.3 ± 10.6	< 0.001
**Vital signs**					
Heart rate (/min)	111.1 ± 28.9	105.2 ± 28.8	111.7 ± 28.6	116.4 ± 28.3	< 0.001
Mean BP (mmHg)	60.0 (50.0, 121.0)	60.0 (50.0, 119.0)	61.0 (50.0, 121.2)	60.0 (49.0, 123.0)	0.794
**Laboratory date**					
WBC(10^9/L)	13.4 (9.7, 18.5)	12.9 (9.4, 17.6)	13.0 (9.8, 18.1)	14.1 (9.7, 20.1)	0.105
Bun(mg/dL)	27.0 (17.0, 43.0)	23.0 (15.0, 33.0)	26.0 (18.0, 43.0)	34.0 (19.0, 53.0)	< 0.001
Cr(mg/dL)	1.3 (0.9, 2.2)	1.1 (0.8, 1.6)	1.4 (1.0, 2.2)	1.6 (1.0, 2.8)	< 0.001
TC(mg/dL)	108.0 (86.0, 139.0)	112.0 (88.0, 143.0)	106.0 (84.5, 133.0)	105.0 (84.0, 142.0)	0.102
TG(mg/dL)	105.0 (71.0, 154.0)	63.0 (51.0, 80.0)	106.0 (84.0, 128.0)	182.0 (134.0, 256.0)	< 0.001
HDL(mg/dL)	31.0(20.0,43.0)	45.0(36.0,54.0)	31.0(24.0,37.0)	16.0(11.0,24.0)	< 0.001
**Sepsis Admission, n (%)**					< 0.001
Sepsis, pulmonary	312 (40.5)	133 (51.8)	94 (36.6)	85 (33.1)	
Sepsis, renal/UTI (including bladder)	200 (25.9)	63 (24.5)	71 (27.6)	66 (25.7)	
Sepsis, GI	83 (10.8)	23 (8.9)	24 (9.3)	36 (14)	
Sepsis, unknown	72 (9.3)	15 (5.8)	29 (11.3)	28 (10.9)	
Sepsis, cutaneous/sof tissue	59 (7.7)	15 (5.8)	23 (8.9)	21 (8.2)	
Sepsis, other	45 (5.8)	8 (3.1)	16 (6.2)	21 (8.2)	
**Severity of illness**					
GCS score	12.6 ± 3.4	13.0 ± 2.9	12.1 ± 3.8	12.5 ± 3.5	0.012
SOFA score	4.1 ± 2.8	3.3 ± 2.4	4.2 ± 2.8	4.6 ± 3.1	< 0.001
**Comorbidities**					
COPD, n (%)					0.104
No	694 (90.0)	223 (86.8)	236 (91.8)	235 (91.4)	
Yes	77 (10.0)	34 (13.2)	21 (8.2)	22 (8.6)	
CHF, n (%)					0.419
No	690 (89.5)	226 (87.9)	229 (89.1)	235 (91.4)	
Yes	81 (10.5)	31 (12.1)	28 (10.9)	22 (8.6)	
AMI, n (%)					0.625
No	706 (91.6)	232 (90.3)	238 (92.6)	236 (91.8)	
Yes	65 (8.4)	25 (9.7)	19 (7.4)	21 (8.2)	
DM, n (%)					0.008
No	645 (83.7)	221 (86)	224 (87.2)	200 (77.8)	
Yes	126 (16.3)	36 (14)	33 (12.8)	57 (22.2)	
Acute kidney injury, n (%)					< 0.001
No	598 (77.6)	221 (86)	207 (80.5)	170 (66.1)	
Yes	173 (22.4)	36 (14)	50 (19.5)	87 (33.9)	

Data are presented as the mean ± SD, median (interquartile range) or percentage. AIP: Atherogenic Index of Plasma; BMI: Body Mass Index; Mean BP: Mean Blood Pressure; WBC:White Blood Cell; Bun:Blood Urea Nitrogen; Cr: Creatinine; TC: Total Cholesterol; TG: Triglycerides; GI:Gastrointestinal; UTI:Urinary Tract Infection; GCS: Glasgow Coma Scale; SOFA: Sequential Organ Failure Assessmen; COPD: Chronic Obstructive Pulmonary Disease; CHF: Congestive Heart Failure; AMI: Acute Myocardial Infarction; DM: Diabetes Mellitus.

### Relationship between AIP and acute kidney injury in patients with sepsis

Table 2 shows the univariate analysis of AKI in sepsis patients. BMI and SOFA score were significantly associated with AKI risk (all P < 0.05). Comorbidities (COPD, CHF, DM) and lipid profiles (TG, AIP) also significantly increased AKI risk, with AIP showing the strongest association (OR=3, P < 0.001). Demographic and hemodynamic factors had no significant effect.

**Table 2:** Unadjusted association between baseline variables and acute kidney injury.

Variable	OR_95 CI	*P* value
Gender		
Male	Reference	
Female	1.15 (0.82 ~ 1.62)	0.407
Age	1 (0.98 ~ 1.01)	0.452
Ethnicity, n (%)		
Caucasian	Reference	
African American	1.07 (0.63 ~ 1.8)	0.807
Hispanic	0.46 (0.22 ~ 0.94)	0.034
Asian	1.08 (0.52 ~ 2.28)	0.833
Native American	^a^	0.973
Other/Unknown	0.54 (0.12 ~ 2.45)	0.426
BMI	1.03 (1.01 ~ 1.05)	0.001
Heart rate	1 (1 ~ 1.01)	0.649
Mean BP	1 (1 ~ 1)	0.858
WBC	1 (0.98 ~ 1.02)	0.782
TC	1 (0.99 ~ 1)	0.232
**Sepsis Admission, n (%)**		
Sepsis, pulmonary	Reference	
Sepsis, renal/UTI (including bladder)	1.23 (0.8 ~ 1.89)	0.35
Sepsis, GI	2.09 (1.23 ~ 3.57)	0.007
Sepsis, unknown	1.08 (0.57 ~ 2.04)	0.806
Sepsis, cutaneous/sof tissue	1.28 (0.66 ~ 2.48)	0.465
Sepsis, other	1.03 (0.47 ~ 2.25)	0.943
GCS score	1.03 (0.98 ~ 1.09)	0.244
SOFA score	1.16 (1.09 ~ 1.23)	<0.001
COPD		
No	Reference	
Yes	1.77 (1.06 ~ 2.95)	0.028
CHF		
No	Reference	
Yes	1.99 (1.21 ~ 3.25)	0.006
AMI		
No	Reference	
Yes	1.61 (0.92 ~ 2.8)	0.095
DM		
No	Reference	
Yes	1.63 (1.07 ~ 2.5)	0.024
AIP	3 (2.06 ~ 4.36)	<0.001

**Fig 2:**
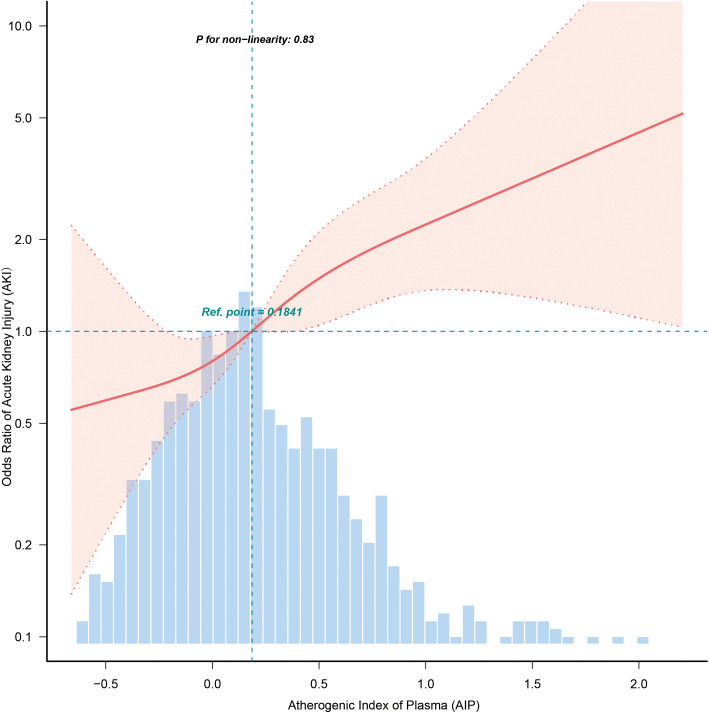
Associations between AIP and AKI in all patients with sepsis. The dose-response relationship between AIP and AKI in septic patients was linear by restricted cubic spline *(RCS) regression analysis. Adjusted for gender, age, ethnicity,* BMI, *Heart rate, Mean* BP, WBC, *TC, Sepsis admission*, GCS *score*, SOFA *score*, COPD, CHF, *AMI and* DM.

Fig 2 showed a statistically significant linear correlation using a smooth curve fitting analysis. Table 3 demonstrates the association between AIP and AKI in sepsis patients. Multivariate analysis showed that for every 0.1 unit increase in AIP (AIP*10), the risk of AKI increased by 10% after adjusting for sex, age, race, BMI, GCS score, SOFA score, heart rate, mean blood pressure, COPD, CHF, AMI, DM, WBC, TC, and sepsis admissions (Model 3: OR = 1.10, 95% CI: 1.05–1.15, P < 0.001). When stratified by AIP, the T3 group (0.364 to 2.204) had a significantly higher risk of ARF compared to the T1 group (Model 3: OR = 2.52, 95% CI: 1.49 to 4.26, P = 0.001), and there was a trend towards a significant dose-response (trend P < 0.001). A sensitivity analysis was conducted to address the concern that adjusting for the SOFA score, which includes a renal component, might introduce overadjustment bias in modeling AKI risk. After removing the SOFA score from the fully adjusted model, the association between AIP and AKI remained robust (see S1 Table).

**Table 3:** Relationship between AIP and acute kidney injury in patients with sepsis.

Variable	Model 1		Model 2		Model 3	
	OR (95%CI)	*P* value	OR (95%CI)	*P* value	OR (95%CI)	*P* value
AIP*10	1.12 (1.07 ~ 1.16)	<0.001	1.12 (1.08 ~ 1.16)	<0.001	1.10 (1.05 ~ 1.15)	<0.001
AIP tertiles						
T1(−0.697 ~ 0.017)	1(Ref)		1(Ref)		1(Ref)	
T2(0.017 ~ 0.363)	1.48 (0.93,2.37)	0.099	1.48 (0.92,2.36)	0.103	1.37 (0.8 ~ 2.33)	0.252
T3(0.364 ~ 2.204)	3.14 (2.03,4.86)	<0.001	3.2 (2.05,4.98)	<0.001	2.52 (1.49 ~ 4.26)	0.001
*P* for Trend		<0.001		<0.001		<0.001

Model 1 No adjusted.

Model 2 adjusted for gender and age.

Model 3 adjusted for gender, age, ethnicity, BMI, Heart rate, Mean BP, WBC, TC, Sepsis admission, GCS score, SOFA score, COPD, CHF, AMI and DM.

AIP*10: AIP (per 0.1 unit).

### Secondary results: Relationship between AIP and length of stay in patients with sepsis

As demonstrated in Table 4, the association between AIP and prolonged hospitalization remained robust across both continuous and categorical analyses. When analyzed as a continuous variable, each 0.1 unit increase in AIP was associated with an additional 0.31 hospital days. Further stratification by tertiles revealed that patients in the highest AIP tertile experienced 2.56 more hospital days compared to those in the lowest tertile.

**Table 4:** Relationship between AIP and length of stay in patients with sepsis.

Variable	Model I		Model II		Model III	
	β (95%CI)	*P* value	β (95%CI)	*P* value	β (95%CI)	*P* value
AIP*10	0.31 (0.17 ~ 0.46)	<0.001	0.27 (0.13 ~ 0.42)	<0.001	0.27 (0.1 ~ 0.44)	0.002
AIP tertiles						
T1(−0.697 ~ 0.017)	1(Ref)				1(Ref)	
T2(0.017 ~ 0.363)	0.59 (−1.01 ~ 2.19)	0.470	0.47 (−1.12 ~ 2.06)	0.565	0.05 (−1.65 ~ 1.74)	0.957
T3(0.364 ~ 2.204)	3.09 (1.49 ~ 4.69)	<0.001	2.76 (1.15 ~ 4.37)	0.001	2.56 (0.78 ~ 4.34)	0.005
*P* for Trend		<0.001		0.001		0.005

Model I No adjusted.

Model II adjusted for gender and age.

Model III adjusted for gender, age, ethnicity, BMI, Heart rate, Mean BP, WBC, TC, Sepsis admission, GCS score, SOFA score, COPD, CHF, AMI and DM.

### Sensitivity analysis

Table 5 presents sensitivity analyses after excluding patients with kidney infections (n = 571). After multivariable adjustment, each 0.1-unit increase in AIP remained significantly associated with acute kidney injury risk (OR = 1.08, 95% CI:1.03–1.14, P = 0.004). The highest AIP tertile (T3) showed 1.98-fold higher risk compared to the lowest tertile (T1) (95% CI:1.09–3.59, P = 0.025), with a significant dose-response trend (P = 0.018). These findings further confirm the independent association between AIP and acute kidney injury.

**Table 5:** Relationship between AIP and acute kidney injury in sensitivity analyses (excluding patients with kidney infection).

Variable	Model 4	
	OR (95%CI)	*P* value
AIP*10	1.08 (1.03,1.14)	0.004
AIP tertiles		
T1(−0.697 ~ 0.017)	1(Ref)	
T2(0.017 ~ 0.363)	1.08 (0.58 ~ 2)	0.809
T3(0.364 ~ 2.204)	1.98 (1.09 ~ 3.59)	0.025
*P* for Trend		0.018

Model 4 was sensitivity analysis after excluding those with patients with kidney infection, n = 571. We adjusted for gender, age, ethnicity, BMI, Heart rate, Mean BP,WBC, TC, GCS score, SOFA score, COPD, CHF, AMI and DM.

As shown in S2 Table, after we reanalyzed the data after multiple interpolation for missing data, the AIP index remained positively associated with the occurrence of acute kidney injury. The results before and after imputation showed consistent trends, which verified the robustness of the study.

### Subgroup analysis

**Fig 3:**
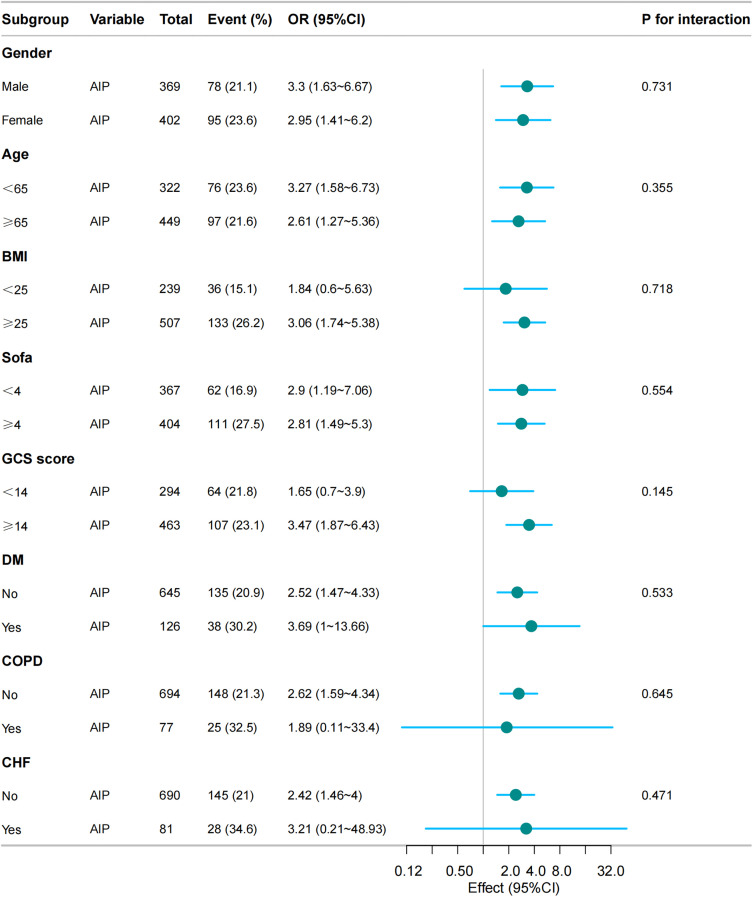
Subgroup analysis of AIP on AKI. *Adjusted Variables: gender, age, ethnicity*, BMI, *Heart rate, Mean* BP, WBC, *TC, Sepsis admission*, GCS *score*, SOFA *score*, COPD, CHF, *AMI, and* DM*, excluding the stratification variables*.

Fig 3 presents subgroup analyses of the association between AIP and acute kidney injury. A positive association was consistently observed across subgroups stratified by gender, age, BMI, disease severity (SOFA score), and comorbidities (diabetes, COPD, CHF). Notably, stronger associations were found in patients with BMI ≥ 25 kg/m² (OR = 3.06, 95% CI:1.74–5.38), GCS score ≥ 14 (OR = 3.47, 95% CI:1.87–6.43), and those with diabetes (OR = 3.69, 95% CI:1.00–13.66). All interaction P values were > 0.05, indicating no significant heterogeneity in the AIP-AKI association across subgroups.

## Discussion

In this large, retrospective cohort study analyzing data from the eICU Collaborative Research Database spanning 208 ICUs across the United States, we identified a strong and independent dose-dependent relationship between elevated AIP and increased risk of AKI in patients with sepsis. The findings showed that each 0.1-unit increment in AIP corresponded to a 10% increase in the odds of developing AKI (OR 1.10, 95% CI 1.05–1.15). Patients in the highest AIP quartile experienced a more than 2.52-fold elevated risk compared to those in the lowest quartile. This association persisted after adjustment for a wide range of confounders including vital signs, severity of illness scores, infection site, comorbidities, and inflammation markers. Therefore, our study confirmed AIP as an independent risk factor for AKI in sepsis patients.

The clinical relevance of these findings is underscored by their consistency with prior evidence linking dysregulated lipid profiles to kidney injury. A 0.1-unit increase in AIP approximates to a roughly 26% increase in the TG/HDL-C ratio, which could result from clinically relevant changes in lipid levels (e.g., an increase in triglycerides or a decrease in HDL-C). Previous research demonstrated that elevated AIP predicted adverse renal outcomes in patients with acute pancreatitis [18] and those with chronic kidney disease [21], though these conditions represent distinct pathophysiologic contexts. Our investigation extends the scope of AIP to the critically ill sepsis population—a group characterized by amplified metabolic disturbances, endothelial dysfunction, and immune activation—further suggesting that lipid metabolism plays a pivotal role in the pathogenesis of sepsis-induced organ damage.

Mechanistically, the observed relationship between high AIP and AKI likely reflects multiple interacting pathways. Elevated AIP corresponds to an atherogenic lipid profile dominated by pro-inflammatory triglycerides relative to anti-atherogenic HDL cholesterol, which promotes lipid accumulation within renal tubular epithelial cells. This lipotoxicity can disrupt mitochondrial function and induce oxidative stress, culminating in cellular injury and apoptosis [30–35]. Additionally, AIP-associated endothelial dysfunction may contribute to decreased renal perfusion and potentiate ischemia-reperfusion injury during sepsis. The correlation of AIP with systemic inflammatory mediators such as CRP and IL-6 indicates a potential synergistic effect whereby lipid dysregulation exacerbates inflammation-driven renal tubular damage through oxidative stress and cytokine overproduction [36–40]. Importantly, our results demonstrated the independence of this association beyond generalized inflammation and established markers of severity, as the relationship persisted after controlling for white blood cell counts and SOFA scores. This supports the hypothesis that AIP captures metabolic derangements distinct from the traditional inflammatory cascade and may provide incremental prognostic value.

The core findings of this study form a significant scientific correspondence with existing literature: A cohort study of 22,952 general population participants from the National Health and Nutrition Examination Survey (NHANES) demonstrated that, after multivariate adjustment, the highest quartile of the AIP was associated with a modestly increased risk of CKD compared to the lowest quartile (OR = 1.24, 95% CI: 1.02–1.52, P = 0.023) [21]. Notably, another study involving 1,470 acute pancreatitis (AP) patients reported that 250 (17%) developed AKI and 166 (11.3%) progressed to severe AP; after adjusting for confounders, AIP showed a significant association with AKI risk (OR = 2.5, 95% CI: 1.31–4.77) [18]. These studies collectively suggest, from perspectives of chronic non-inflammatory nephropathy and localized inflammatory diseases, that AIP may serve as a shared biomarker for inflammation-related organ damage. For the first time in a sepsis-induced multiple organ dysfunction syndrome (MODS) population, this study rigorously controlled for confounders and confirmed an independent positive correlation between AIP and AKI.

Strengths of the study: First, we demonstrated for the first time the correlation between AIP and AKI in patients with sepsis. Second, we took into account confounding factors to the greatest extent possible. Third, we conducted sensitivity analyses to assess the reliability of the findings. These analyses included converting AIP into a categorical variable, reassessing the relationship between AIP and AKI by excluding individuals whose initial site of infection was the kidney, and performing multiple imputations to further demonstrate the stability of the results. Additionally, subgroup analyses were performed, and no significant interactions across subgroups were detected.

Limitations of the study: Firstly, despite adjusting for multiple variables in the retrospective design based on the electronic database, it remains challenging to completely control for residual confounding factors (such as unmeasured genetic susceptibility, the status of chronic kidney disease before sepsis, use of lipid-lowering medications, and detailed nutritional status). Consequently, the causal temporal relationship between AIP and AKI cannot be established, and a prospective cohort study is needed for further verification. Secondly, the outcome definition primarily captures AKI within 48 hours of ICU admission, and may miss later-onset cases. Thirdly, the study only included the baseline AIP value. However, during the course of sepsis, changes in the inflammatory response, nutritional interventions, and organ function can significantly affect lipid metabolism. Therefore, it is necessary to dynamically monitor the trajectory of AIP in the future to explore its association with the progression of renal injury. Finally, potential misclassification of sepsis or AKI due to the reliance on administrative coding should be considered. The eICU database, from which the data were sourced, predominantly consists of the North American population, which introduces geographical and ethnic biases. The universality of AIP as a biomarker requires external validation in different populations in Asia, Europe, and other regions.

Our study also aligns with emerging research on related biomarkers, such as the lactate/albumin ratio and blood urea nitrogen to albumin ratio, which have shown prognostic value in critically ill populations with AKI and sepsis. These findings highlight the importance of metabolic and nutritional parameters as critical contributors to risk stratification and outcome prediction in critical illness. Integrating AIP with such indices could further refine predictive models and individualized clinical decision-making.

In conclusion, this study provides robust evidence that elevated AIP is independently associated with an increased risk of AKI in patients with sepsis, underscoring its potential utility as a prognostic biomarker for early risk stratification. Understanding the mechanistic links between lipid metabolism, inflammation, and renal injury in sepsis is crucial for developing targeted interventions to mitigate renal complications. Future well-designed prospective studies, including serial AIP monitoring and multi-ethnic populations, are warranted to validate and extend these findings and to explore therapeutic implications.

## Conclusions

To summarize, AIP is associated with acute kidney injury in septic patients. These findings suggest that routine surveillance of AIP can be a valuable clinical tool for early identification of patients at risk for AKI. By identifying those at risk, healthcare providers can implement early interventions and personalised care strategies, ultimately improving patient prognosis.

## Supporting information

S1 Table Sensitivity analysis of the association between AIP and Acute kidney injury with and without adjustment for SOFA score. Model 1 adjusted for gender, age, ethnicity, BMI, Heart rate, Mean BP, WBC, TC, Sepsis admission, GCS score, COPD, CHF, AMI and DM. Model 2 adjusted for gender, age, ethnicity, BMI, Heart rate, Mean BP, WBC, TC, Sepsis admission, GCS score, COPD, CHF, AMI and DM,SOFA score.
(DOC)

S2 Table Relationship between AIP after multiple imputation and Acute kidney injury in patients with sepsis. Model 1 No adjusted. Model 2 adjusted for gender and age. Model 3 adjusted for gender, age, ethnicity, BMI, Heart rate, Mean BP, WBC, TC, Sepsis admission, GCS score, SOFA score, COPD, CHF, AMI and DM.
(DOC)
