# Supplementary material for: Association between the atherogenic index of plasma and acute kidney injury in sepsis patients
Source: PLoS One. 2026 Mar 10;21(3):e0344477. doi: 10.1371/journal.pone.0344477 (PMC12974842; doi:10.1371/journal.pone.0344477)
Supplement: S1 Table — Model 1 adjusted for gender, age, ethnicity, BMI, Heart rate, Mean BP, WBC, TC, Sepsis admission, GCS score, COPD, CHF, AMI and DM. Model 2 adjusted for gender, age, ethnicity, BMI, Heart rate, Mean BP, WBC, TC, Sepsis admission, GCS score, COPD, CHF, AMI and DM,SOFA score. (DOC) [file pone.0344477.s001.doc]

**S1 Table. Sensitivity analysis of the association between AIP and Acute kidney injury with and without adjustment for SOFA score**

| Variable | Model 1  OR (95%CI) *P* value | | Model 2  OR (95%CI) *P* value | |
| --- | --- | --- | --- | --- |
| AIP*10 | 1.12 (1.07~1.18) | <0.001 | 1.10(1.05~1.15) | <0.001 |
| AIP tertiles |  |  |  |  |
| T1(-0.697~0.017) | 1(Ref) |  | 1(Ref) |  |
| T2(0.017~0.363) | 1.51 (0.9~2.55) | 0.103 | 1.37 (0.8~2.33) | 0.252 |
| T3(0.364~2.204) | 3.09 (1.86~5.13) | <0.001 | 2.52 (1.49~4.26) | 0.001 |
| *P* for Trend |  | <0.001 |  | <0.001 |

Model 1 adjusted for gender, age, ethnicity, BMI, Heart rate, Mean BP, WBC, TC, Sepsis admission, GCS score, COPD, CHF, AMI and DM.

Model 2 adjusted for gender, age, ethnicity, BMI, Heart rate, Mean BP, WBC, TC, Sepsis admission, GCS score, COPD, CHF, AMI and DM ,SOFA score.
