# Supplementary material for: Association between the atherogenic index of plasma and acute kidney injury in sepsis patients
Source: PLoS One. 2026 Mar 10;21(3):e0344477. doi: 10.1371/journal.pone.0344477 (PMC12974842; doi:10.1371/journal.pone.0344477)
Supplement: S2 Table — Model 1 No adjusted. Model 2 adjusted for gender and age. Model 3 adjusted for gender, age, ethnicity, BMI, Heart rate, Mean BP, WBC, TC, Sepsis admission, GCS score, SOFA score, COPD, CHF, AMI and DM. (DOC) [file pone.0344477.s002.doc]

**S2 Table. Relationship between AIP after multiple imputation and Acute kidney injury in patients with sepsis**

| Variable | Model 1 | Model 2 | Model 3 |
| --- | --- | --- | --- |
| AIP*10 | 1.12 (1.07, 1.16) <0.0001 | 1.12 (1.08, 1.16) <0.0001 | 1.11 (1.06, 1.16) <0.0001 |
| AIP tertiles |  |  |  |
| T1(-0.697~0.017) | 1 | 1 | 1 |
| T2(0.017~0.363) | 1.48 (0.93, 2.37) 0.0993 | 1.48 (0.92, 2.36) 0.1027 | 1.32 (0.80, 2.19) 0.2839 |
| T3(0.364~2.204) | 3.14 (2.03, 4.86) <0.0001 | 3.20 (2.05, 4.98) <0.0001 | 2.55 (1.56, 4.16) 0.0002 |
| *P* for Trend | <0.0001 | <0.0001 | 0.0001 |

Model 1 No adjusted

Model 2 adjusted for gender and age

Model 3 adjusted for gender, age, ethnicity, BMI, Heart rate, Mean BP, WBC, TC, Sepsis admission, GCS score, SOFA score, COPD, CHF, AMI and DM .
